# Extracorporeal shockwave lithotripsy for lower pole calculi smaller than one centimeter

**DOI:** 10.4103/0970-1591.44260

**Published:** 2008

**Authors:** Christian Chaussy, Thorsten Bergsdorf

**Affiliations:** Department of Urology, Klinikum Harlaching, Staedtisches Klinikum, Muenchen GmbH, Germany

**Keywords:** Extracorporeal shockwave lithotripsy, lower pole, nephrolithiasis

## Abstract

Extracorporeal shockwave lithotripsy (ESWL) has revolutionized the treatment of urinary calculi and became the accepted standard therapy for the majority of stone patients. Only for stones located in the lower calix, ESWL displayed a limited efficacy. Since the stone-free rate seemed to be preferential, endoscopic maneuvers like percutaneous nephrolithotomy (PCNL) and retrograde intrarenal surgery (RIRS) have been proposed as the primary approach for this stone localization.

Stone size seems to be the most important parameter in regard to the stone-free rate, whereas anatomical characteristics of the lower pole collecting system are discussed controversial. Various studies show a good stone clearance between 70-84% for stones up to 1 cm in diameter. Additional physical and medical measures are suitable to improve treatment results. Stone remnants after ESWL, defined as clinical insignificant residual fragments (CIRF) will not cause problems in every case and will pass until up to 24 months after treatment; in total 80-90% of all patients will become stone-free or at least symptom-free.

When complete stone-free status is the primary goal, follow-up examinations with new radiological technologies like spiral CT show that the stone-free rate of ESWL and endoscopically treated patients (RIRS) does not differ significantly. However, in comparison to endoscopic stone removal, shockwave therapy is noninvasive, anesthesia-free and can be performed in an outpatient setup. Therefore, ESWL remains the first choice option for the treatment of lower caliceal stones up to 1 cm. The patient will definitely favour this procedure.

Since its introduction in 1980[1] extracorporeal shockwave lithotripsy (ESWL) became the preferred treatment option for the majority of renal calculi because of its noninvasive nature and low potential of complications. But there was always a controversial debate whether lower pole stones are a good target for ESWL therapy. A meta-analysis, published by Lingeman in 1994[2] and a number of publications in the following time showed a reduced efficacy of ESWL for lower pole calculi. Meanwhile, the design of modern flexible endoscopes with a deflection up to 270° made lower pole stones accessible via ureteroscopy and enabled treatment of those stones with a high primary success rate.

Nevertheless, there are many good arguments to give ESWL the preference for the treatment of lower pole stones, especially if the stone burden is low.

Long-term studies of different authors indicate a cumulative stone free rate of 41-73% for lower pole stones. [2–7] When the treatment results are stratified in respect to stone size, various studies show a good stone clearance between 70-78% for lower caliceal stones up to 10 mm.[1,7–9,10] Second and third generation lithotripters with their proposed less disintegration efficacy in comparison to the unmodified DORNIER HM 3 seem to achieve higher stone-free rates. Robert *et al.*, described a stone-free rate of 84% for lower caliceal stones between 5 and 15 mm with piezoelectric ESWL.[11] A reason might be the smaller size of fragments which can be achieved with modern lithotripsy systems; therefore the passage of fragments is facilitated and results in an improved passage of stone fragments.

The disintegration rate of lower caliceal stones treated by ESWL is comparable to stones in other localizations within the kidney after ESWL. However, due to the unfavourable spatial anatomy of the lower pole collecting system, the clearance of the fragments is not as likely. Sampaio[12] analyzed 1992 different characteristics of the lower pole as possible influencing factors for the passage of stone debris. Several succeeding studies[13–16] described the influence of the lower pole infundibulopelvic angle (LIPA), the infundibulum length and infundibulum width in respect to the stone-free rate. An acute lower pole infundibulopelvic angle, a tight infundibular width and a long infundibular length are associated with a reduced stone-free rate.[16] In contrast, Ather[17] and Sorensen[18] found no statistically relevant interference of lower pole anatomy on stone-free rate; the only relevant parameter was stone size.[19] Knoll *et al.*,[20] concluded that the high interobserver variation of measuring the specific features of the lower pole anatomy were responsible for these inconsistent published data.

The most important contribution to a good stone clearance is the stone disintegration into small, sand-like fragments. Small stone remnants have a higher likelihood to pass even collecting systems with an unfavorable spatial anatomy. Newer generation lithotripters (electromagnetic, piezoelectric) with their smaller focal geometry and reduced energy settings seem to achieve a finer stone disintegration, but need more often repetitive treatments for complete fragmentation.[10,11] This so called “BOOSTER-strategy” achieves higher stone-free rates. In our own data, the overall stone-free rate for lower caliceal stones after three months was, with 70%, slightly higher than the stone-free rates for middle and upper caliceal stones (65% vs. 67%).

A further aspect is physical therapy, like mechanical percussion, inversion therapy and diuresis, to assist the passage of lower pole stone fragments after shockwave therapy.[21,22] In a randomized, prospective study, Chiong *et al.*, reported a significantly higher stone-free rate for patients with a combination of ESWL plus PDI (percussion, diuresis, inversion) in comparison to ESWL therapy alone (62,5% vs. 35,4% stone-free rate). Subsequent medical treatment after ESWL therapy may be beneficial to improve stone-free rate and decrease recurrence. Micali *et al.*,[23] achieved an increased stone-free rate for patients with a regular self-administration of Uriston (Phyllanthus niruri) after shockwave therapy. After six months, the patient collective with medication reported a significantly higher stone-free rate of 93,7% in comparison to medicamentous untreated patients with 70,8%. The efficacy of metaphylaxis with potassium citrate in calcium oxalate stone formers was underlined by a randomized controlled trial of Soygur *et al.*[24] In patients who were stone-free after ESWL and medical treatment, the stone recurrence rate after 12 months was 0%, whereas untreated patients showed a 28,5% recurrence rate. Similarly, in the residual fragment group, the medicamentous treated patients had a significantly higher remission rate than the untreated patients (44,5% vs. 12,5%).[25] Some authors[26,27] used a direct irrigation of the lower pole collecting system via a percutaneous nephrostomy or a cystoscopically placed cobra catheter, to wash out stone fragments during ESWL. Nicely *et al.*, reported an increase of stone-free rate to 71% in comparison to 54% without irrigation. But this strategy transforms a noninvasive therapy option (ESWL) into a more invasive procedure with additional risks and complications.

Additional physical therapy and medication are suitable to improve the treatment results of lower pole stones. But there remain some interesting questions:

- What is the definition of “stone-free”?
- What is the optimal diagnostic tool to estimate stone-free status?
- What is the adequate time interval for control?
- Is it really necessary to get every patient stone-free?

The literature gives no clear consensus for the definition of “stone-free”. Only the complete absence of stone remnants after treatment should be considered as “stone-free”, but some authors include patients with stone fragments less than 5 mm, so-called clinical insignificant residual fragments (CIRF), into this group. A further controversial debate continues for the suitable diagnostic modality, to consider the patient stone-free. [28–33] The plain abdominal X-ray (KUB) is accepted as the first line diagnostic method for follow-up examination after stone therapy, but in most cases it overestimates the stone-free rate. Küpeli *et al.*, found in their study, that ultrasound will find stone remnants in 11,8% and helical computed tomography in 22,3% of patients, who are considered stone-free in plain abominal X-ray.[28] Another aspect is the inter-observer (52%) and intra-observer variation (24%) of radiological findings in plain X-rays, reported by Jewett *et al.*[29] Non-contrast spiral CT seems to be the most sensitive radiological tool for the detection of residual fragments after stone therapy,[30–32] but is associated with a higher radiation dosage for the patient in comparison to conventional X-ray. Follow-up examinations with CT after endoscopic stone removal disprove the excellent treatment outcomes shown initially on plain abdominal X-ray. Park *et al.*,[30] found stone-free rates after percutaneous nephrolithotomy (PCNL) of 62,3% in a KUB control. The stone-free rate dropped to 20,8% when the examination was performed with a non-contrast CT. Similar findings were reported by Portis *et al.*,[32] for the treatment of upper urinary tract calculi by means of ureteroscopic laser lithotripsy. 54% of the patients were judged stone-free in the one month follow-up with non-contrast spiral CT.

Residual fragments (CIRF) after stone treatment were not only a topic for ESWL-treated patients; endoscopic procedures like ureteroscopy (URS) and PCNL leave patients with residual fragments behind as well. In a prospective, randomized study, comparing shock wave lithotripsy (SWL) and URS for lower pole caliceal calculi 1 cm or less, Pearle and Lingeman[33] found no statistical significance in the stone-free rate between SWL and URS (35% vs. 50%) in the three months follow-up with spiral CT and concluded that SWL was associated with greater patient acceptance and shorter convalescence.

For this reason, some authors avoid the term “stone-free” and prefer instead the term “treatment success”, summarizing stone-free patients and patients with residual fragments less than 5 mm size. It is acceptable to call residual fragments up to 4 mm a treatment success, because these CIRF show remarkable dynamics. Stone-free rate at three months follow-up will not display the definite treatment outcome. Long-term studies showed a continuous stone passage of stone fragments up to 24 months after shockwave therapy.[34,35] Osman *et al.*, evaluated patients with a mean follow-up time of 4,9 years, who have been treated by ESWL and released with CIRF. In 78,6% CIRF cleared spontaneously and did not reappear within five years.[36] Summing up, CIRF are a typical consequence of modern stone therapy, but 80-90% of all patients become stone-free or asymptomatic with CIRF.

This are many good arguments for ESWL treatment of lower pole calculi smaller than 1 cm in diameter – but is this the procedure of first choice? The guidelines of various urological societies (AUA, EAU, DGU) recommend ESWL as first choice for the treatment of lower pole caliceal stones up to 1 cm. Gerber[37] asked 205 urologists about their choice of treatment for lower caliceal stones. The preferred approaches were ESWL for stones < 1 cm and PCNL for those > 2 cm. For stones of 1 to 2 cm, 65% preferred SWL and 30% would recommend PCNL.

And do not forget: if the patient has the choice he will mostly prefer the least invasive therapy option.

## CONCLUSION

Extracorporeal shockwave lithotripsy is the first choice for the treatment of lower pole caliceal stones up to 1 cm and favored by urologists and patients because it is the only noninvasive therapy option and can be performed without anesthesia in an outpatient setup. Long-term radiological follow-up shows no significant difference in stone-free rate in comparison to retrograde intrarenal surgery (RIRS) but is associated with less significant complications, faster convalescence and greater patient acceptance.
